# Chemical cannibalistic cues make damselfly larvae hide rather than hunt

**DOI:** 10.1038/s41598-023-40732-2

**Published:** 2023-08-21

**Authors:** Monika Sysiak, Barbara Pietrzak, Matylda Kubiak, Anna Bednarska, Andrzej Mikulski

**Affiliations:** Department of Hydrobiology, Institute of Functional Biology and Ecology, Faculty of Biology, University of Warsaw, Żwirki i Wigury 101, 02-089 Warsaw, Poland

**Keywords:** Ecology, Evolution, Physiology, Zoology, Ecology, Limnology

## Abstract

Adopting cannibalism substantially affects individual fitness, and recognizing the presence of other cannibals provides additional benefits such as the opportunity to prepare for hunting or defense. This recognition can be facilitated by perceiving conspecific chemical cues. Their role in cannibalistic interactions is less studied than in interspecific predation and it is unclear whether these cues inform individuals of danger or of food availability. Interpretation of these cues is crucial to balance the costs and benefits of anti-predator and feeding strategies, which can directly influence individual fitness. In this study we aimed to test whether damselfly larvae shift towards bolder and more exploratory (cannibalistic) behavior, or become more careful to avoid potential cannibals (as prey) in response to such cues. We conducted behavioral and respiratory experiments with *Ischnura elegans* larvae to investigate their response to chemical cues from older and larger conspecific larvae. We found that *I. elegans* larvae decrease their activity and shift their respiratory-related behavior, indicating activation of anti-predator defense mechanisms in response to conspecific chemical cues. Our findings indicate that individuals exposed to conspecific chemical cues balance catching prey with staying safe.

## Introduction

Cannibalism is a widespread phenomenon^1–3^ of intraspecific predation and as such it has consequences to population dynamics. It eliminates potential conspecific competitors, especially important at high population densities^4^ or when resources are scarce. Cannibalism is often size or density dependent and can affect phenology, size and age structure of the population, and lead to its self-regulation^5–7^.

Cannibalistic individuals may enhance their fitness through nutritional benefits, yet are exposed to additional risks. Well-fed cannibals increase their own survival, developmental rate, and fecundity^8–10^. In caddisfly *Asynarchus nigriculus* larvae inhabiting temporary wetlands, cannibalism helps cope with temporal constraints by shortening larval period and enhances imago's fitness^11^. Despite benefits, cannibalistic behavior might have negative consequences. Pathogens or parasites can be horizontally transmitted when infected conspecifics are consumed, or the cannibal may assimilate hormones, other active compounds, or bioaccumulated toxins from the body of its prey^12–14^. Moreover, cannibals risk injury or even death from defensive responses of their potential prey^5^. On the other end, potential prey risks life and thus benefits greatly from the ability to recognize and respond to cannibalistic danger.

The effectiveness of strategies employed by prey to evade predators relies on the accessibility and precision of sensory data. While vision theoretically allows for immediate perception of predator's location, approach trajectory, and identity, prey confronted with active predators must react so early that visual cues become unreliable indicators of real danger^15^.

Therefore, in predator–prey interactions, recognizing each other’s presence via chemical cues is one of the most effective and reliable mechanisms of assessing either risk or prey availability. Such cues may be of one of three types: alarm signal, diet cues, or kairomones. Infochemicals released by injured individuals form alarm signal which serves as cue of danger to other potential prey. Diet cues, released by hunted prey and produced during prey consumption and expulsion by predators, provide information to other predators about food availability. Lastly, kairomones are involuntarily released by both prey and predator and betray each other's presence without the necessity of direct interaction^16^.

In interspecific predation, predator’s reaction to the chemically-detected presence of prey is often limited to behavioral changes in foraging strategy^16^.The adaptive response of prey, on the other hand, is often complex and includes changes of its behavior^17^, life history^18^, and morphology^19^. This requires precise control of which mechanism to activate when perceiving danger, as the choice must account for associated costs and potential trade-offs^20^. Prey response largely depends on the kind of the signal, e.g., alarm signal can elicit stronger prey response than kairomones^21^, but also the concentration of kairomones (consequence of predator density) may determine the strength of prey reaction^22,23^. The importance of aforementioned chemical cues for interspecific predation is vastly exemplified^24–26^.

The role of these chemicals in intraspecific, cannibalistic interactions is more complex and far less understood. Most of the hitherto conducted studies on cannibalistic interactions concern the effect of conspecific alarm signal, while kairomones have been studied much less in this respect. For example, blue crabs (*Callinectes sapidus*) avoid areas of intense conflicts in response to odors released from freshly injured conspecifics. Yet, alarm signal can also function as diet cues in this intraspecific interaction^27^. Hermit crabs (Paguroidea) respond to conspecific chemical cues, but instead of triggering defense mechanisms, they increase foraging intensity^28^. Chemical communication is here involved in the key evolutionary dilemma of balancing the trade-off between costs and benefits associated with anti-predator behavior and feeding, as an individual perceiving conspecific cues may either adopt the role of a predator (risky but profitable) or of a prey (safe but costly due to induced defense mechanisms) and it is little known how this is decided. The cannibalistic strategy involves pursuing prey to acquire nutrient benefits, while the prey strategy is to avoid risk of being eaten by conspecifics at the cost of potential fitness decrease. One can thus expect an individual to change behavior to bolder, more exploratory and aggressive in order to find and catch conspecific prey, or to more careful to avoid potential cannibals. Inducible response to conspecific chemical cues should thus directly lead to either improvement (when an individual adopts predator-cannibal strategy) or reduction (when an individual adopts prey strategy) of foraging performance.

Odonate larvae are a suitable model for investigating the importance of chemical cues in cannibalistic interactions. Larval cannibalism has been found in different odonate species^29^ and it might be so prevalent at initial instars as to impact population more than interspecific predation. In the later stages, cannibalism in damselfly larvae may have a competitive basis beyond mere nutrient provision. For instance, cannibalism in *Megaloprepus coerulatus* occurs in larvae fed ad libitum, and decreases when the population density falls down to about one larva per 1.5 liter^30^. Cannibalism in odonates is common throughout the larval period, affecting growth rate, body mass, or the timing of emergence^31,32^. Also, the way the larvae hunt may cause conspecific signals to be perceived as danger. They are mid-ranking predators, abundant in the littoral zone, often among macrophytes, using either a sit-and-wait or a climber (slowly following the prey) strategy, and attack only when the danger and time required to manipulate the prey are low^33^. When all conspecific larvae are hidden in refuge or are lurking for prey, visual and mechanical cues do not provide enough information about the upcoming attack. Under such conditions, conspecific chemical cues may provide reliable information about the threat from foraging larvae. Thus, conspecific chemical cues of other zygopteran larvae may convey information about the risk of being attacked by a hiding cannibal.

Behavioral changes are a common anti-predator strategy in zygopteran larvae and alterations to foraging are expected as the response to conspecific cues. For example, a typical anti-predator defense response in damselfly larvae is reduction in foraging intensity (decreasing the number of captured prey) and prolonging immobility^34^. This reduces the risk of being noticed by predator^20,35^. When predator is present, zygopteran larvae often use refuge, e.g. macrophytes, to hide^36^. Thus, predator presence can substantially influence individual behavioral patterns.

Predator pressure can also lead to changes in respiratory-related behavior, thus causing changes in the oxygen uptake pattern, which we call here oxygen consumption variability. Stressed zygopteran larvae modify the pattern of rectal breathing by increasing the number of inhalations, which shortens the pause between them and intensifies exhalation^37^. Intensified inhalation may be a way to take in more oxygenated water. Intensified exhalation, i.e. ejection of water, can mix water between lamellae (three leaf-like multi-functional organs used for gas exchange, during swimming, and sacrificed if grasped by a predator^38^), supplying oxygenated water. This mechanism can be valuable when predator presence results in reduced prey mobility and the larva is limited in performing movements that promote gas exchange, such as swimming^39^. Moreover, oxygen demand may vary depending on the severity of the threat and the prey's defense strategy. Oxygen consumption of prey can increase^40–42^ or decrease under predation risk^43,44^. Lowered respiration rates often correspond to reduced prey activity^44^. Higher respiration rates may be observed during short-term behavioral response as individuals attempt to escape^41,42^. Thus, if an individual adopts a prey strategy by reducing its mobility, a decrease in respiratory rate is likely to occur, but there may be an increase in oxygen consumption variability. On the other hand, if the individual adopts a cannibalistic predator strategy, an increase in respiratory rate and a decrease in oxygen consumption variability can be expected. Thus, parameters related to oxygen consumption provide additional information on the strategy adopted by the individual.

Here, we conducted behavioral (mobility and feeding) and respiratory experiments with larvae of damselfly *Ischnura elegans* to test if they: (a) change their behavioral strategy in response to conspecific chemical cues by reducing mobility and staying in refuge (i.e. adopt prey strategy), (b) decrease oxygen consumption and change oxygen consumption pattern by increasing its consumption variability in response to those signals (i.e. adopt prey strategy), and if (c) the strength of the individual reaction to conspecific cues depends on their type and concentration.

## Materials and methods

### Experimental animals

Damselfly larvae *Ischnura elegans* were collected from Czerniakowskie lake in Warsaw—a natural urban lake inhabited by both fish and invertebrate predators—in July 2020. Larvae were photographed and measured using NIS-Elements software, and assigned to instars according to parameters proposed by Thompson: body length and head width^45^. Additionally, the instar assignment was confirmed by the visual inspection of the stage of wing bud development. Individuals were classified into three groups: (1) cannibals—11th and 12th instar, (2) prey (of cannibals)—7th and 8th instar, (3) experimental animals (individuals in which reaction to chemical cues was observed)—10th instar. The width of the zygopteran larva's head limits the upper size of the prey it can hunt. Individuals classified to 11th and 12th instars had heads wider than those in 10th instar by 50 and 75%, respectively. Thus, the differences in size between the individuals classified into these two groups were sufficient for the cannibals (11th and 12th instars) to be able to prey on the experimental individuals (10th instar). Hence, the kairomones of the oldest larvae should be a reliable signal of a potential threat.

All experimental animals were synchronized to the 10th instar. Knowing the date of the larva's last molt allowed us to choose the time of the experiment to exclude unexpected molting during exposition to kairomones. Individuals in the 9th instar were kept in specially-designed containers (material: colorless filament ASA 1.75 mm, dimensions: 50 × 70 × 40 mm) created with 3D printing technology that guaranteed the physical separation of individuals. The aquarium with the containers was filled with 10 L of water. There were 10 individuals per aquarium. The culturing setup consisted of a series of interconnected chambers that were separated by a mesh, enabling a continuous flow of fresh, oxygenated water through each individual chamber. The aquarium contained a designated vegetation zone (to prevent the accumulation of excess nitrogen compounds), wherein a pump was located. The pump functioned to expel water from the vegetation zone to the opposite end of the aquarium, thereby maintaining a consistent flow of oxygenated water throughout the entirety of the system.

Every second day, the odonates were fed with live adult *Daphnia pulex*. After reaching the 10th instar, the larvae were transferred to a thermostatic chamber and each was kept in a separate container (to prevent cannibalistic interaction and exposition to conspecific cues), at 4 °C, the low temperature preventing them from molting. The cannibals (11th and 12th instar larvae) and prey (7th and 8th instar larvae) were also maintained at 4 °C. Additionally, as an injury may affect larvae behaviors and respiratory activities, all experimental animals were checked for signs of mechanical damage (such as loss of lamellae or legs). All larvae were cultivated in conditioned (filtered through 1 µm mesh and aerated at least for two weeks) lake water.

### Media preparation

Before the experiment, all cannibals were acclimatized to 20 °C for three days. These damselflies were not fed prior to the experiment 1) to prevent contamination of their kairomones with food particles that could serve as potential alarm signal (AS) for prey, and 2) to increase their propensity for cannibalism. A day before the experiment, the predators were transferred to separate containers—to prevent competitive or cannibalistic interactions—where they were exposed in conditioned lake water for 24 h to produce kairomones.

The experimental media were as follows: 1) Control medium—kairomone free medium (C); 2) Medium with kairomones in concentration of 2 *I. elegans* × L^−1^ (D2)—reflecting low population density, thus lower risk of cannibalism. To obtain it, each of the cannibals was exposed separately in 500 ml of clean medium; 3) Medium with kairomones in concentration of 5 *I. elegans* × L^−1^ (D5)—reflecting high population density, thus higher risk of cannibalism. Here, each of the cannibals was exposed separately in 200 ml of clean medium; 4) Medium containing both kairomones in high concentration and conspecific alarm signal, i.e. chemicals released by prey injured by the cannibals (D5 + AS)—reflecting high population density, thus higher risk of cannibalism (as D5), with the additional alarm signal (AS) informing about the presence of an actually foraging predator. The procedure was similar to the preparation of D5 medium, yet cannibalism was allowed among odonates in order to obtain conspecific AS. The smaller prey individual was placed in a container with an individual that produced kairomone only for the last two hours of exposition in order to prevent alterations to the concentration of kairomones. It was a one-to-one interaction. Each cannibal, in its separate container, ate one prey.

To prepare experimental media, the replicated cannibal-exposed water from all containers used in the particular treatment was poured into a single one just before the experiment and filtered through a GFC filter.

### Behavioral (mobility and feeding) experiment

The experiment was performed in a specially designed chamber (white box of dimensions 120 × 106 × 56 cm) which guaranteed stable internal conditions and silenced off external noises. The box was equipped with two lamps covered with a light diffuser to ensure uniform light conditions. It was placed in the temperature-controlled experimental room and special openings provided gravity ventilation, thus allowing it to maintain the constant 20 °C inside. The camera was inserted through a hole in the center of the box's ceiling. The front wall was removable, allowing an easy access to the animals.

**Figure 1:**
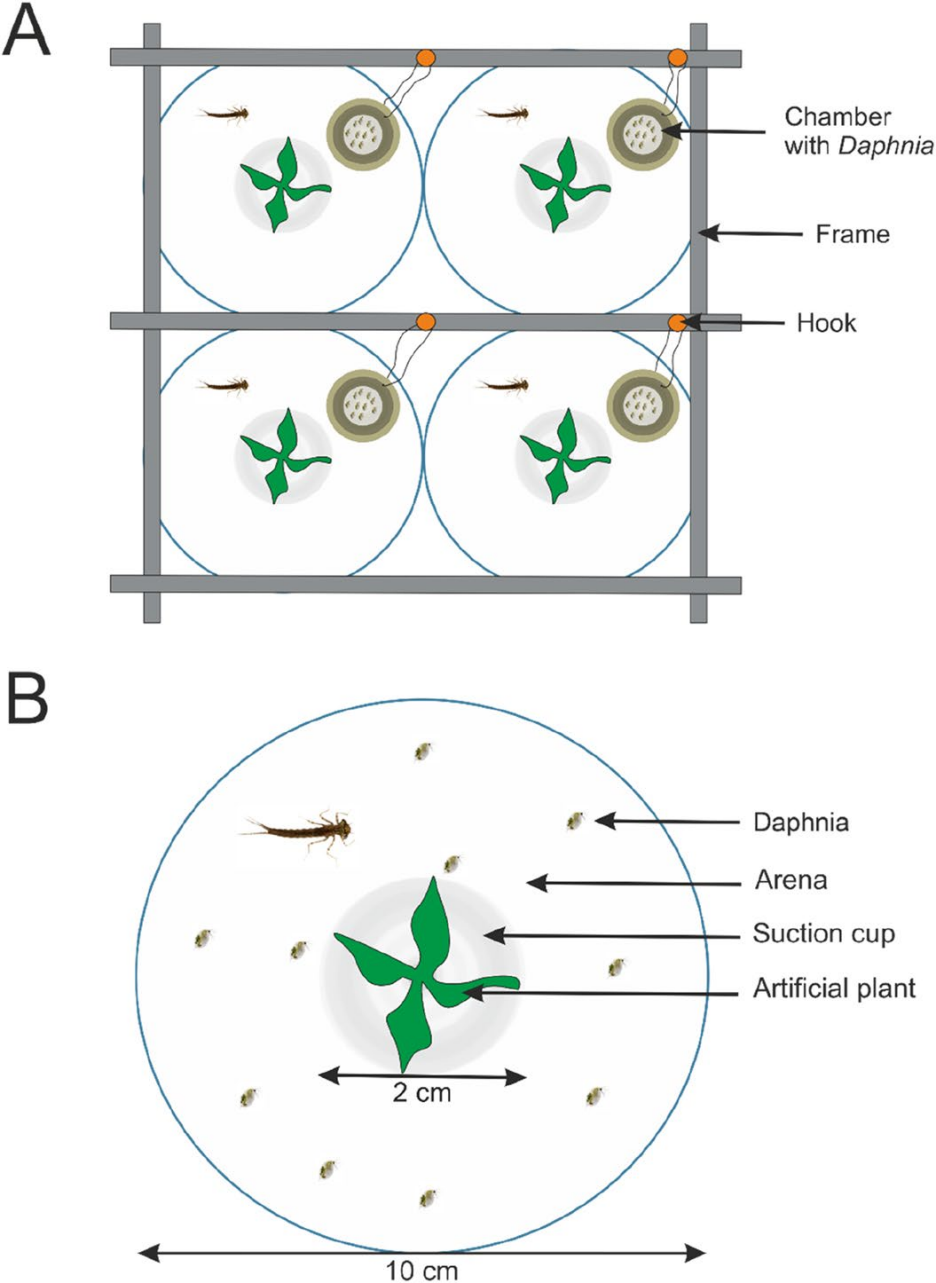
Experimental setup. (**A**) Feeding device with cylindrical bottomless chambers holding *Daphnia* while resting on the bottom of the experimental containers, four containers in sight. The chambers were tied to hooks on the rack (gray) and lifted during feeding, (**B**) An individual experimental container with arena zone and refuge zone (artificial plant on suction cup) with *Daphnia* released.

Six days before the experiment, experimental animals were transferred from 4 to 20 °C for acclimatization. Each of them was placed in a separate container to prevent exposure to conspecific chemical cues and cannibalistic interactions. The next day after the transfer, the animals were moved into the box in their separate containers. These were round, non-transparent plastic vessels (diameter 10 cm, height 3 cm) filled with 200 ml of clean medium and had a centrally located refuge zone (an artificial plant on a silicone suction cup of 2 cm in diameter,—Fig. 1). The circular shape of the containers minimized the shaded area inside and thus increased the clarity of the recorded movies. Additionally, it prevented the aggregation of live food in the corners and enforced its random dispersion. Each experimental animal was fed with 10 *D. pulex* at the pre-reproductive instar. Live food was introduced simultaneously into the containers with *I. elegans* larvae with the use of a special feeding device (Fig. 1A) that consisted of a movable rack to which containers with live *Daphnia* were attached. When the rack was lifted, the *Daphnia* were released into the containers with *I. elegans*. Three days before the experiment, the medium was exchanged and damselflies were fed as before. Then, the larvae were starved for two days prior to the experiment.

On the experimental day the containers were filled with 200 ml of one of the experimental media. The control medium (C) and the medium with high kairomone concentration with an alarm signal (D5 + AS) were replicated six times. The medium with low kairomone concentration (D2) and the medium with high kairomone concentration (D5) were replicated five times (this unequal number of replications resulted from problems with collecting a sufficient number of synchronized experimental individuals). The larvae were then left to acclimatize for 30 min to reduce the impact of the stress from transferring to experimental containers on mobility and feeding behaviors. After acclimatization, the feeding procedure was performed. After closing the chamber, the recording of feeding behavior was started and lasted for 63 min.

### Movie analysis

Before the analysis, the initial three minutes were cut from the raw recording (starting from the moment of closing the box), as in that time larvae might have shown altered behavior caused by retraction of the feeding device and movements associated with the closing of the box. An hour-long recording was used for the analysis. To compare the behavior of odonate larvae at the beginning (immediate response to cue—we assume that in this time the effect of kairomones is most detectable) and at the end of this period, the movie's first and last 15 min were analyzed. Each larva was analyzed independently. Nine types of behaviors were observed and coded with three letters. The first letter denotes where the behavior takes place, in refuge (R) or in arena (A). The second letter denotes whether the animal is immobile (I) or mobile (M). The third letter after the hyphen specifies further either the location of the activity: on plant (-P) or on cup (-C), or the type of mobility: leaning (-L), treading (-T), gentle movements (-G) or swimming (-S). The behaviors were: 1) RI-P: Immobility on plant—most camouflaging behavior; 2) RI-C: Immobility on suction cup—observation of prey; 3) RM-P: Mobility on plant (vertical movement)—stalking of prey; 4) RM-C: Mobility on suction cup (horizontal fast walking)—stalking of prey; 5) RM-L: Leaning out in search of prey—stalking of prey**;** 6) AI: Immobility within arena—observation of prey; 7) AM-G: Gentle movements within arena (turning the head behind the prey, slow initial walking)—initial stalking of prey; 8) AM-T: Treading within arena—(decisive, quick following of the prey) main stalking of prey; 9) AM-S: Swimming—quick escape. Behavior timeline was created separately for each individual's first and last 15 min of the recording.

From the timeline of individuals assigned to each treatment (i.e. different infochemicals), the first and last 15 min of experiment were taken and two parameters were determined for each treatment for each period: i) time spent immobile (sum of times spent on RI-P, RI-C, AI behaviors), ii) time spent in refuge (sum of times spent on RI-P, RI-C, RM-P, RM-C, RM-L behaviors). We chose these parameters expecting that movement and switching between different types of behavior may attract predator. Thus, time spent in refuge, and time spent immobile should be a good measure of fear of a cannibal predator. We analyzed the data for the first and last 15-min intervals separately using TIBCO Statistica 13.3 and Kruskal–Wallis test (results of Leven’s and Shapiro–Wilk test showed that data failed to meet the criteria for parametric analysis) and a nonparametric two-tailed post-hoc test with Bonferroni correction described by Castellan, with significance level at *p* < 0.05^46^.

Also, the total number of attacks on prey made in the first and last 15 min of the experiment was counted. We counted all attack attempts, including those that were not successful. An attack was considered an ejection of the mask in the direction of the prey. Simultaneously, we also recorded the success of the attack, which involved mask ejection leading to the capture of the prey. We analyzed the data for the first and last 15-min intervals separately. Additionally, at the end of the experiment the number of *Daphnia* left in each experimental container was counted to obtain the number of prey consumed during the whole experiment. All three parameters: 1) total number of attacks, 2) number of successful attacks, and 3) number of consumed *Daphnia* until the end of the exposure were analyzed using TIBCO Statistica 13.3. and Kruskal–Wallis test (data failed to meet the criteria for parametric analysis) and nonparametric two-tailed nonparametric post-hoc test with Bonferroni correction described by Castellan with significance level at *p* < 0.05^46^.

Second, to analyze the overall effects of different chemical cues on time spent in different behaviors, but also on time in immobility and in refuge, we fitted linear models using generalized least squares by maximizing the restricted log-likelihood (nlme R package^47^). Repeated measurements for individual larvae (first and last 15-min periods) were considered in the error structure and type-II analysis-of-variance table was calculated for these models (Anova function of car package^48^). To calculate pairwise comparisons between groups with correction for multiple testing we used pairwise Wilcoxon rank sum tests with Holm’s method of *p*-value adjustment^49^. All these calculations were performed using the R language and environment^50^. In addition, we qualitatively describe the observed behavioral patterns.

We visualized behavioral patterns for the first and last 15 min in each treatment as directed graphs. Each of the nine observed behavioral types was represented on a graph as an ellipse (bubble) corresponding in area to the total time spent on this behavior by all individuals from the particular treatment. The ellipse area was calculated as log (time) * 10. The number of direction-specific transitions between behaviors for all individuals from each treatment was visualized by arrow thickness, which was calculated as log (number of transitions*2) * 1.5. The graphs were created in CorelDRAW2020.

### Respiratory experiment

The media used in respiratory experiments were prepared as described above, except that a 0.2 μm antibacterial filter was used for media filtration before the proper experiment, and next, the proper medium was aerated for 15 min.

On each day of the experiment, only one replication of one of the four experimental treatments was carried out (one individual per day). This makes a total of twelve experimental days, with three repetitions per treatment throughout the entire experiment. The selection of a day for each treatment tested was random. Two days before testing, one experimental animal was transferred from 4 to 20 °C. On the day before testing, an *I. elegans* larva was placed in 200 ml of conditioned and filtered (GFC) lake water. During that day, the larva was starved to empty its digestive tract. The exchange of water was conducted two hours before the experiment.

The oxygen content was measured with a UNICENSE MicroRespiratory System. Two breathing chambers were filled with 40 ml experimental medium (one with the animal and one blank), filtered with a 0.2 μm filter. In the next stage, the filled breathing chambers were placed in a stand located in a water bath set at 20 °C. The blank chamber was measured to account for unexpected oxygen consumption. The oxygen content in each chamber containing an animal was measured continuously for 12 h (with 1 s intervals between measurements) starting from the acclimatization period of 40 min after closing. Oxygen loss at that time ranged between 5 and 27%, not causing the alerting behavior of *I. elegans* associated with oxygen deficiency, which could disturb the hunting process. The volume of each chamber and the animals' body mass was measured after the experiment.

### Respiration data analysis

We analyzed the effect of experimental treatment on damselfly respiration separately for four consecutive three-hour intervals. Since the linear regression parameters changed over time, dividing the analysis into four time intervals increased the precision of deviations from the trendline analysis, see below. To obtain the initial and final oxygen concentration for each of the periods, the measurements for the first and last 15 min were averaged (to neutralize the effect of short-term fluctuations in measurements) and converted per unit mass, by taking into account the body mass of the particular animal, and time. In the blank chambers the measurements did not indicate any unexpected oxygen consumption, hence blank chambers were not included in the analysis.

The variability of oxygen consumption derived from calculating the average deviation from the regression curve calculated separately for each animal. Raw data were log transformed and analyzed by ANOVA and LSD post-hoc test.

## Results

### Time spent immobile

**Figure 2:**
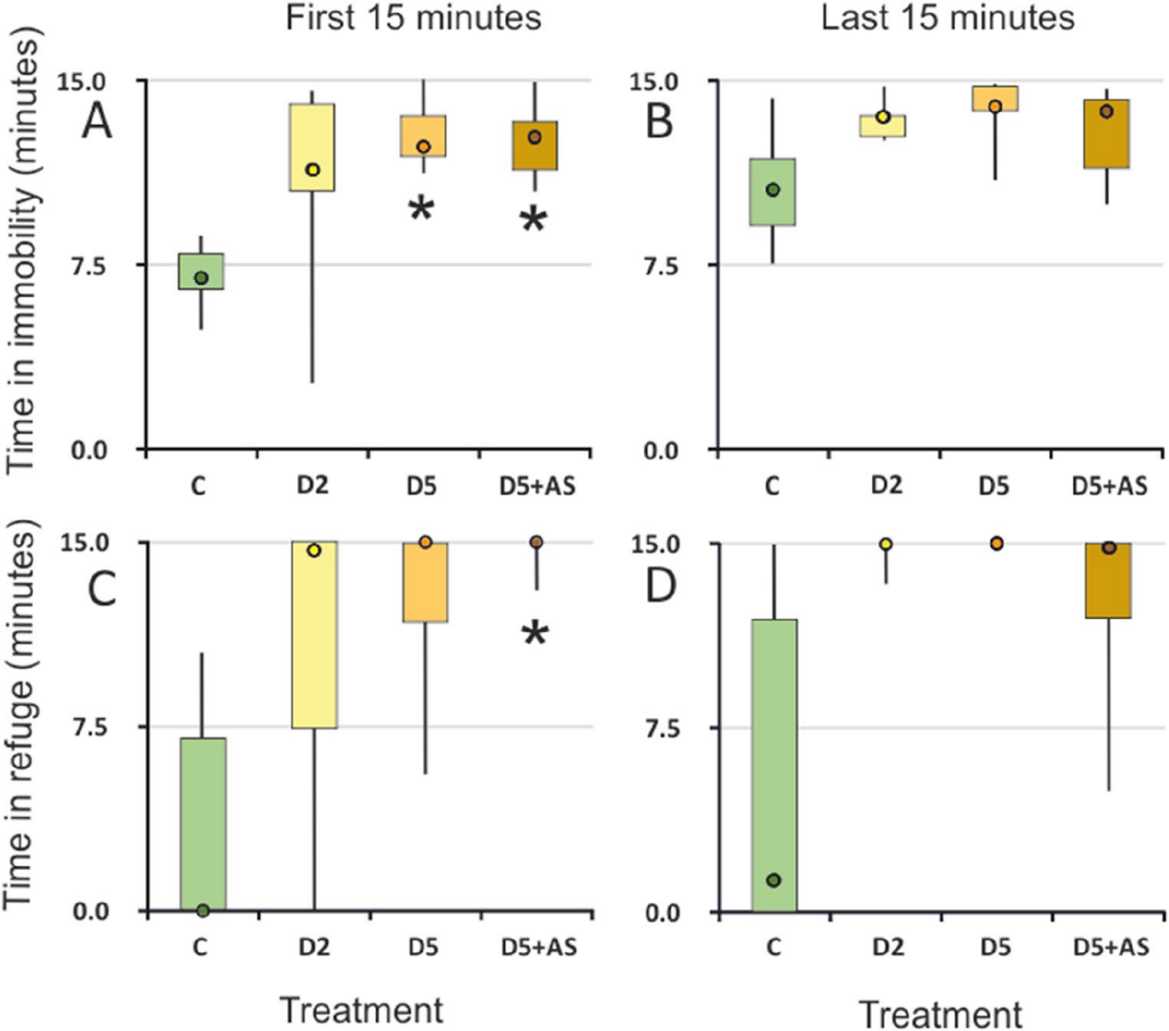
Time spent immobile (**A**, **B**) and time spent in refuge (**C**, **D**) by *I. elegans* exposed to different concentrations of kairomones (C, D2, D5) or kairomones with alarm signal (D5 + AS); Median (circle), 1st and 3rd quartiles (box), min/max (whiskers), *denotes treatments significantly different from the control treatment, C.

In the first 15 min of observation, conspecific kairomones had a significant effect on time the experimental individuals spent immobile (Kruskal–Wallis test: H (3, 22) = 10.45; *p* = 0.021). D5 (high kairomone concentration) and D5 + AS (high kairomone with alarm signal) exposed larvae stayed immobile longer than control larvae (Castellan’s test: *p* = 0.033 and *p* = 0.042, respectively, Fig. 2A). In the last 15 min, the effect was insignificant (Kruskal–Wallis test: H (3, 22) = 7.66, *p* = 0.05, Fig. 2B).

### Time spent in refuge

In the first 15 min, conspecific kairomones with alarm signal had a significant effect on time spent in refuge (Kruskal–Wallis test: H (3, 22) = 10.43, *p* = 0.015). D5 + AS larvae stayed longer in refuge than control larvae (Castellan’s test: *p* = 0.01, Fig. 2C). In the last 15 min, the effect was insignificant (Kruskal–Wallis test: H (3, 22) = 6.16, *p* = 0.104, Fig. 2D)**.**

### Number of attacks

**Figure 3:**
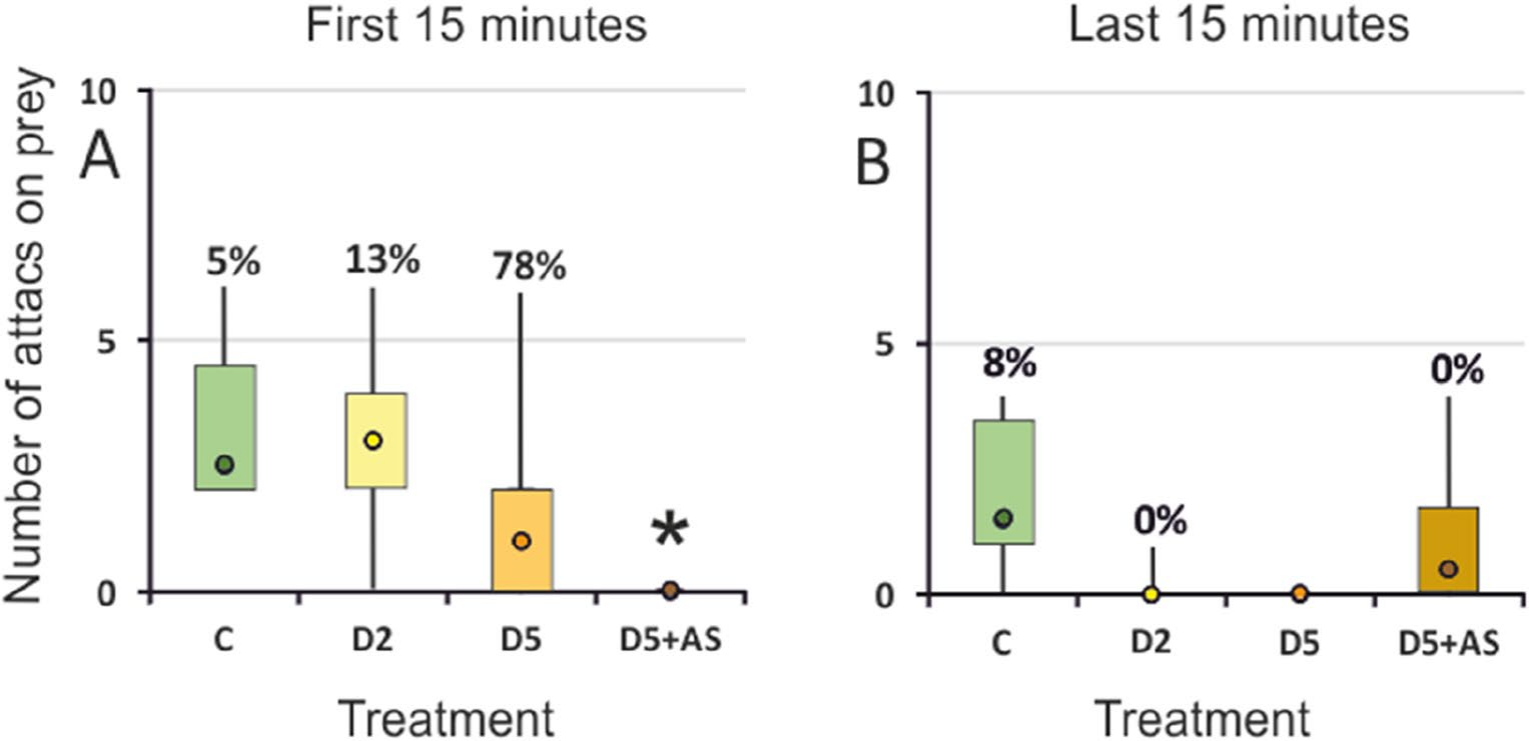
Total number of attacks on *Daphnia* by *I. elegans* exposed to different concentrations of kairomones (C, D2, D5) or kairomones with alarm signal (D5 + AS) in the first 15 min (**A**), and the last 15 min (**B**) of the experiment; Median (circle), 1st and 3rd quartiles (box), min/max (whiskers); Percentages of successful attacks are given over the whiskers; *denotes treatments significantly different from the control treatment (C).

In the first 15 min, there was a significant effect of conspecific kairomones with alarm signal on the total number of attacks (Kruskal–Wallis test: H (3, 22) = 10.96, *p* = 0.012, Fig. 3A). D5 + AS larvae made significantly fewer attacks than control individuals (Fig. 3A). Moreover, as the concentration of kairomones increased, attacks tended to be less frequent but more effective. In the last 15 min, the effect was insignificant (Kruskal–Wallis test, H (3, 22) = 8.86, *p* = 0.031, Fig. 3B).

### Number of consumed *Daphnia*

**Figure 4:**
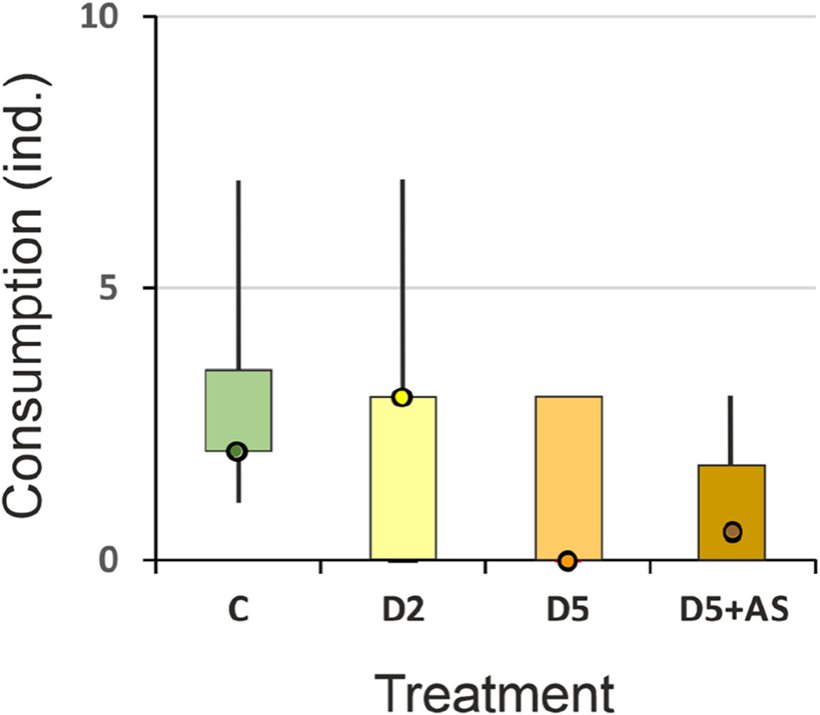
Number of *Daphnia* consumed by *I. elegans* exposed to different concentrations of kairomones (C, D2, D5) or kairomones with alarm signal (D5 + AS); Median (circle), 1st and 3rd quartiles (box), min/max (whiskers).

The number of consumed *Daphnia* was not significantly different between treatments (Kruskal–Wallis test: H (4, 27) = 6.42, *p* = 0.17). However, control animals tended to consume more prey than those from D5 and D5 + AS treatments (Fig. 4).

### Time spent in different behaviors, immobility, and refuge during the whole analyzed time

The presence of conspecific chemical cues had a significant effect on time spent in immobility on suction cup (RI-C), on immobility, slow movements and treading within arena (AI, AM-M, AM-T), and on total time spent immobile and in refuge (Anova on GLS results: Chi^2^ > 12.9, df = 3, *p* < 0.001; Table 1). In the presence of any cues the larvae spent less time immobile within arena, more time immobile overall, and more time in refuge than in control (*p* < 0.05, Wilcoxon pairwise comparisons with Holm correction). Under high concentration of kairomones (D5), they spent more time immobile on cup and less time moving slowly or treading within arena than in control. For slow movements in arena this effect was also seen under alarm signal (D5 + AS).

**Table 1:** Analysis of deviance table (type II tests) for linear models fitted using generalized least squares (GLS) and *p* values for pairwise comparisons with control treatment using Wilcoxon rank sum test with Holm *p* value adjustment for time *I. elegans* exposed to different concentrations of kairomones (C, D2, D5) or kairomones with alarm signal (D5 + AS) spent in different behaviors, immobility, and refuge during the whole analyzed time.

Behavior	df	χ^2^	*p*	D2	D5	D5 + AS
RI-P	3	3.74	0.291	0.300	1.000	0.420
RI-C	**3**	**12.99**	**0.005**	0.126	**0.007**	0.079
RM-L	3	1.77	0.622	0.650	1.000	0.710
RM-P	3	2.18	0.535	0.680	0.110	0.110
RM-C	3	2.50	0.476	1.000	0.820	1.000
AI	**3**	**24.24**	**< 0.001**	**0.030**	**0.016**	**0.016**
AM-G	**3**	**15.68**	0.001	0.106	**0.031**	**0.024**
AM-T	**3**	**15.01**	**0.002**	0.127	**0.024**	0.070
AM-S	3	1.85	0.605	1.000	0.920	1.000
Immobility	**3**	**21.19**	**< 0.001**	**0.045**	**0.002**	**0.009**
Refuge	**3**	**29.63**	**< 0.001**	0.054	**0.008**	**0.008**

Designation of letters in behavioral abbreviations: *R* Refuge, *A *Arena, *I* Immobility, *M* Mobility, *P* Plant, *C* Suction cup, *L* Leaning, *T* Treading, *G* Gentle, *S* Swimming. Immobility and Refuge—total time spend immobile or in refuge.

Significant values are in bold.

### Behavioral patterns

#### Control larvae

**Figure 5:**
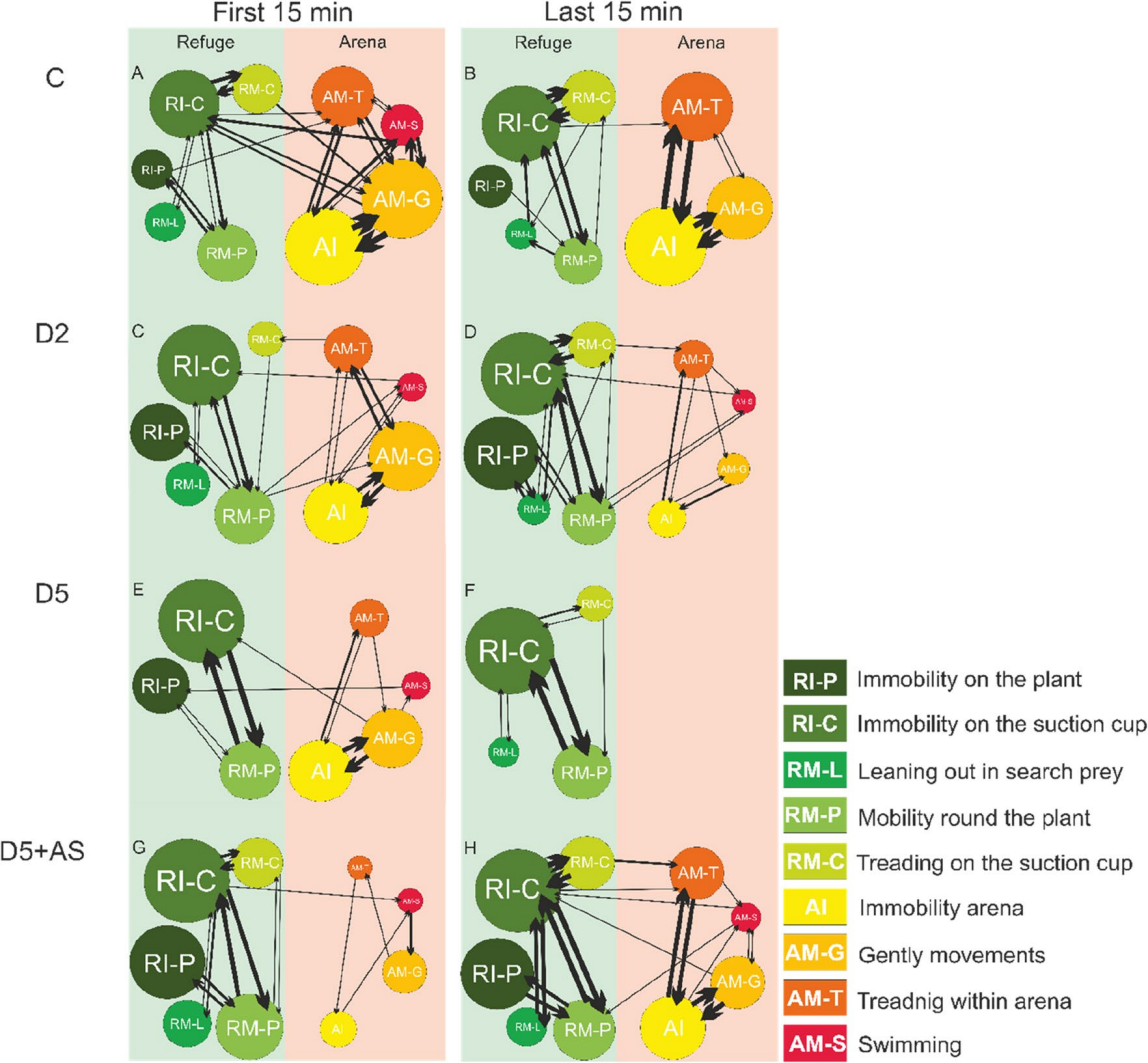
Behavioral patterns of experimental *I. elegans* larvae in each of the four treatments: C (**A**, **B**), D2 (**C**, **D**), D5 (**E**, **F**) and D5 + AS (**G**, **H**). The size of the bubble is proportional to the time spent on the behavior. The thickness of the arrow is proportional to the number of transitions between behaviors. Green colors indicate behaviors considered safe (in refuge), and red-yellow colors are for ones considered dangerous (in the open arena) for the odonates.

In the first 15 min, control individuals displayed all behaviors quite evenly (Fig. 5A). They mostly stayed immobile or gently moved through the arena (AI and AM-M), and least often leaned out in search of prey (RM-L) or trod on suction cup (RM-T) in refuge. Behavioral transitions were frequent and occurred almost between all types of behaviors, yet often between immobility and moving forward. The arena-refuge boundary was often crossed. In the last 15 min (Fig. 5B), the diversity of the behavioral transitions decreased and the arena-refuge boundary was crossed only sporadically.

#### D2 larvae

The behavioral pattern of larvae exposed to information on low population density of conspecifics (D2, Fig. 5C) was similar to the control pattern, though it was less movement-intense and the differences between first and last 15 min were less pronounced. The larvae transitioned between many different behaviors, yet they spent more time immobile in refuge (RI-C, RI-P) than control ones. The arena-refuge boundary was crossed a few times. In the last 15 min (Fig. 5D), D2 individuals spent less time in the arena than in the first 15 min.

#### D5 larvae

The behavioral pattern of larvae exposed to information on high population density (D5), in the first 15 min (Fig. 5E) appeared to be simplified compared to the control. Stalking behaviors (RM-L and RM-T) were not observed. The behavioral pattern was mainly limited to transitions between immobility on cup and mobility in plant in refuge (RI-C and RM-P) and between immobility and gentle movements in the arena (AI and AM-G). The former were more frequent and the latter less frequent compared to control. The arena-refuge boundary was crossed sporadically. In the last 15 min (Fig. 5F), D5 individuals stayed in refuge and their behavioral pattern was almost exclusively limited to the above refuge transitions (between RI-C and RM-P).

#### D5 + AS individuals

The larvae exposed to information on high population density with alarm signal (D5 + AS), in the first 15 min (Fig. 5G) spent most of the time immobile in refuge. As above, most transitions occurred between immobility on cup and mobility in plant (RI-C and RM-P), but transitions between mobility on cup and leaning out were also included (RM-C and RM-L). The refuge-arena boundary was crossed only at times. In the last 15 min (Fig. 5H), mobility within refuge increased. Also, the use of the arena increased. There, the transitions took place between immobility and gentle movements or treading (AI and AM-G or AM-T). The refuge-arena boundary was crossed a few times.

### Oxygen consumption and its variability

**Figure 6:**
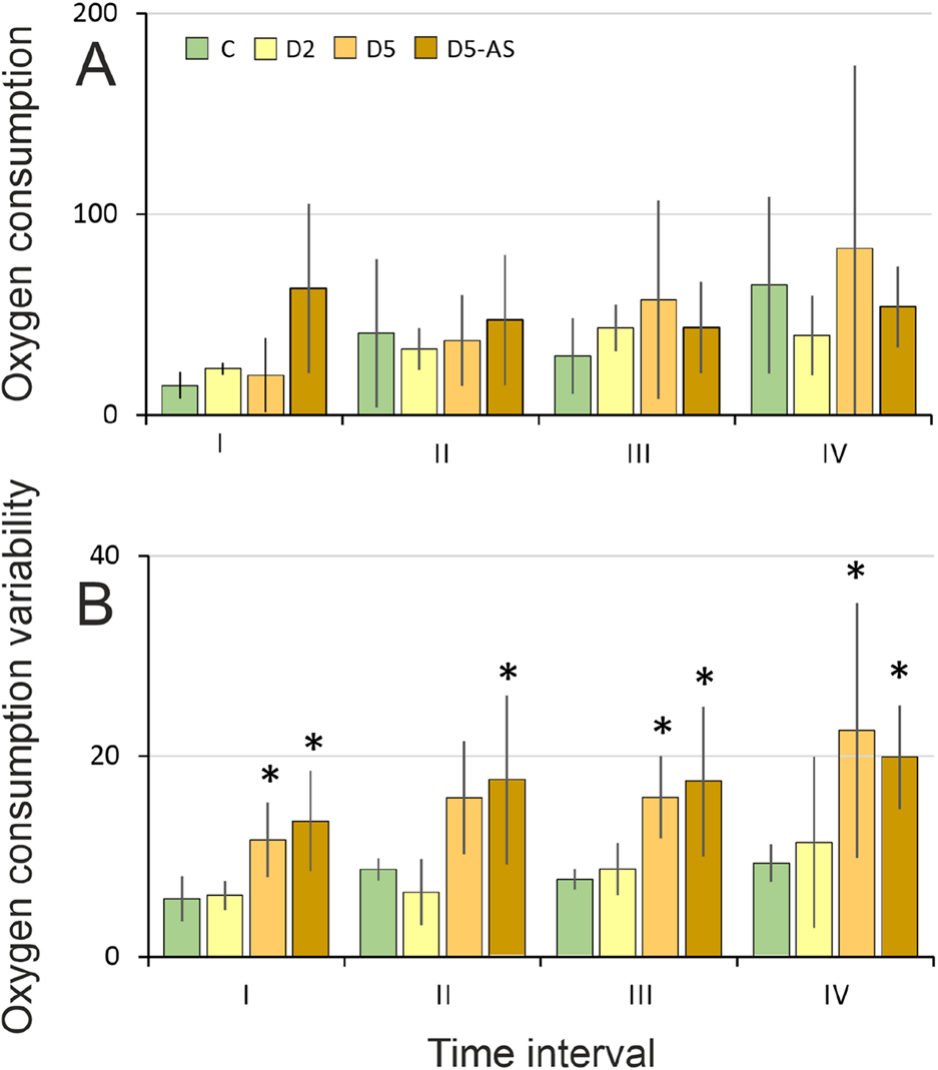
Mean oxygen consumption (O_2_ μmol/ individual mass (mg)) (**A**), and mean oxygen consumption variability (**B**) of *I. elegans* exposed to different concentrations of kairomones (C, D2, D5) or kairomones with alarm signal (D5 + AS). I–IV consecutive time intervals lasting three hours each. The bars indicate standard deviation. * denotes treatments significantly different from the control treatment (**C**) according to LSD (*p* < 0.05).

Oxygen consumption of experimental larvae was not significantly different between treatments in any of the four analyzed time intervals (ANOVA time: F (3, 32) = 2.79, *p* = 0.065, treatment: F (3, 32) = 1.25, *p* = 0.311, Fig. 6A).

A significant effect of time and conspecific cue type on oxygen consumption variability was observed (ANOVA time: F (3, 32) = 3.48, *p* = 0.033, treatment: F (3, 32) = 15.23, *p* < 0.001, Fig. 6B), however, the length of the exposition had no significant effect (*p* = 0.066). D5 individuals had greater variability than control during the first, third and fourth time intervals (I *p* = 0.02, III *p* = 0.002, IV *p* < 0.001, Fig. 6B). D5 + AS larvae showed higher variability of oxygen consumption than control larvae across all analyzed time intervals (I *p* = 0.011, II *p* = 0.001, III *p* = 0.001, IV *p* < 0.001, Fig. 6B).

## Discussion

The role of chemical cues in cannibalistic interactions is more complex than in interspecific predation, yet, it is less understood. Most studies focused on the effects of conspecific alarm signal (AS, i.e. compounds released by injured individuals^16^) and indicated that these cues are perceived either as information about danger or as information about food availability. Less is known about the effects of conspecific kairomones (i.e. compounds released by the predator^16^).

We approached this gap and demonstrated that *I. elegans* odonate larvae decrease their activity in response to conspecific chemical cues—both kairomones and kairomones with alarm signal (Fig. 2A). This indicates recognizing those cues as danger and triggering anti-predator defense, since decreased activity was observed previously in the presence of interspecific predators^51,52^. The choice of the strategy reflects the trade-offs between the need to catch prey and the need to stay safe by decreasing activity and increasing refuge use (Fig. 5). Moreover, changes in behavioral strategies were accompanied by respiratory-related behavior shifts (Fig. 6), which compensated for the restriction of the gas exchange-enhancing active behaviors like swimming.

The behavioral experiment demonstrated that *I. elegans* larvae can recognize conspecific kairomones, as indicated by increased time spent immobile and increased refuge use (Table 1) in kairomones-exposed individuals compared to control. The reaction to kairomones was significant immediately after their application, i.e., within the first 15 min of exposure. (Fig. 2). Reduced foraging activity (Fig. 3) and increased refuge use lower the risk of being detected and thus can serve as predator avoidance strategy^53,54^. Alarm signal also served as a cue of predation danger rather than information about food availability (diet cue), as individuals exposed to kairomones with alarm signal similarly reduced their foraging activity (Table 1), especially at the beginning of exposition (Fig. 2C), and decreased the total number of attacks (Fig. 3A).

Such a reaction to conspecific chemical cues (kairomones and AS) can be related to the odonate larvae's ambush-hunting strategy. They usually remain immobile on habitat structures while waiting for an apparent visual or mechanical stimulus (i.e. rapid movement) from potential prey, after which they either begin to slowly approach the prey or wait for it to come closer by itself^33,55–57^. For surviving as prey, when conspecific chemical cues (implying direct threat from cannibal but not allowing to pinpoint its exact location) are present in the environment, the first behavior—active pursuit of potential prey—may lead to an over-exposure to attack by potential cannibal. For acting as cannibal, chemical cues trigger predatory response only in conjecture with aforementioned mechanical and visual stimuli. Without them, the potential cannibal is unable to assess the size of its conspecific and thus to i.a. judge the chances of winning the fight and not falling prey to cannibalistic interaction. Here we show in *I. elegans* larvae, conspecific kairomones and alarm signal serve predominantly as information about danger rather than prey availability.

Alternatively, the change in foraging strategy in response to conspecific chemical cues may also result from the expected increase in competition. For example, damselfly larvae, during an encounter with a competitor, exhibit aggressive postures and strike each other with their labium until one individual swims away. Such aggressive behavior incurs metabolic costs, which can subsequently affect growth rates^58,59^. Therefore, in some cases, it may be advantageous to "wait out" the presence of a competitor to avoid expending energy needlessly. Thus, the reduction in activity might be also attributed to competition avoidance rather than predator evasion.

The risk of cannibalistic interaction increases with population density^60^. Therefore, conspecific chemical cues indicating population density should be a reliable proxy for the intensity of cannibalistic predation. An animal usually chooses the optimal foraging strategy, which balances the degree of risk with the possibility to accumulate sufficient resources for reproduction. Yet, our research did not evidence changes in behavioral strategy proportional to the degree of threat. We observed only differences in the usage of specific behaviors when comparing the treatment group to the control group (Table 1, Fig. 5).

The behaviors of individuals exposed to low density of conspecifics (D2), indicating low risk of dying due to cannibalistic predation, were similar to behaviors in control. Yet, these individuals appeared to stay more in refuge and immobile (Table 1). The frequent transitions from immobility to gentle movements or treading in both control and D2 individuals indicate a 'stop-and-go strategy' (actively following the prey)^61^, yet, D2 were also immobile on cup and plant just as often. Immobility on cup behavior ensures safe observation of potential prey from refuge, which may be followed by an attack when the prey comes close (‘sit-and-wait’ strategy) or immobility on cup is switched to mobility on plant (‘climber’ strategy—slowly following prey using habitat structures). By extending observation time in refuge, the larvae could assess the prey's speed and distance more accurately, allowing for a more precise pursuit also in the arena. These indications of 'stop-and-go strategy' are consistent with findings of Gilbert^61^, who showed that predator's movements confound continuous detection of prey. Duration of stops is inversely related to prey visual angular velocity—as the prey image moves between neighboring ommatidial fields, the predator relocalizes it and then renews pursuit. This strategy is a compromise between the tracking speed and the ability of the predator's nervous system to process information to assess the velocity and location of prey^61^. The increase in hunting precision of D2 individuals would limit the excessive, unnecessary, violent movements that could be a stimulus for cannibal attacks. It seems that hunting in the stop-and-go strategy was supplemented by infrequent hunting in refuge. The benefit of hunting in refuge involves reducing the distance to prey and decreasing the likelihood of being detected during attack^33,62^, as sudden movements are confined to shelter. The costs include limiting hunting opportunities outside refuge, which may make meeting energetic demands a challenge. A predator may exhibit a combination of the two types of hunting: ambush and active search of prey^63,64^ and both physiology and environment influence the choice of individual foraging behavior^65^. The sit-and-wait strategy brings more energetic benefits when prey density is high. When prey densities decrease, the strategy is switched to active searching for prey^66,67^. In this study, control individuals chose an active foraging strategy forced by low *Daphnia* density during the experiment. Thus, this strategy was most likely the most energy efficient under these experimental conditions. D2 individuals were switching between two foraging tactics: hunting in refuge, safer but less effective, and hunting in arena, riskier but more efficient. The contribution of the two strategies was balanced enough that, as a result, the low density of cannibals did not affect the overall efficiency of D2 larvae foraging (Fig. 4).

The behaviors of individuals exposed to high kairomone concentration (D5), indicating high probability of death due to cannibalism, differed from the control. D5 individuals balanced between hunting in refuge and arena, however, towards more time spent immobile in refuge and less time mobile in arena compared to control group (Table 1). Our results suggest that D5 individuals also simplified their behavioral pattern (Fig. 5E). The perceived predation pressure here was strong enough to make the more efficient arena-hunting less profitable than in C, well visible especially shortly after receiving the cue. Also the percentage of successful attacks seemed highest in this experimental group (78% vs.  ≤ 13% in other treatments, though not statistically significant).This, combined with observations of the behaviors, indicates that these larvae attacked almost only when catching the prey was most likely successful (Fig. 3A). Still, they consumed fewer *Daphnia* during the experiment than control individuals (Fig. 4).

D5 + AS individuals, i.e. exposed to cues of high mortality risk indicated not only by high population density but also by actively foraging cannibals, did not balance between arena and refuge behaviors and chose only safe low-mobility foraging in refuge. They spent more time in refuge and immobile, than the control group. (Table 1), which was evident in the first 15 min of the experiment (Fig. 5G), when the larvae did not attack even once (Fig. 3A). Similarly to *I. elegans* responses observed here, feeding and respiratory changes of *Daphnia magna* studied as prey were also strong when they were exposed to a mixture of kairomones from predators and alarm cues from conspecifics^21^. The strategy of D5 + AS individuals here lined towards bolder behaviors in the last 15 min (Fig. 5H). The larvae may have been inclined to risk more to compensate for the effects of the previous inactivity and refraining from hunting. A fresh alarm signal informs prey that the predator may still be very close, and the older the signal, the more likely the predator has moved away. In line with that, in the study of Barnes^68^, wolf spiders *Paradosa milvina* distinguished between fresher and older predation chemical signal and modified their response accordingly to the severity of the perceived threat.

The results of the respiratory experiment showed no significant effect of conspecific chemical cues on *I. elegans* oxygen consumption (Fig. 6A). Also Kolar found no increased respiration rate under predator pressure in *Ischnura* larvae^69^. The lack of impact may be due to a change in respiratory-related behavior serving as compensatory behavior and resulting from the anti-predation strategy. The quantity of oxygen consumed remained constant, whereas the method of its delivery was altered. High concentration of conspecific kairomones and kairomones with alarm signal caused changes in respiration-related behaviors indicated by higher fluctuations of oxygen consumption rate than in control (Fig. 6B). D5 and D5 + AS larvae spent more time immobile than control ones and thus, unlike controls, were limited to using dissolved oxygen available only in the immediate surroundings of their body. Eriksen showed zygopteran larvae perform several movements promoting increased respiration efficiency, from subtle movements of the abdomen, which mix the water around the lamellae, to moving to new locations where oxygen is not depleted^39^. The apparent modifying of the rectal breathing pattern of D5 and D5 + AS larvae compensated for the inability to make other movements which increase respiration efficiency but can be a visual stimulus for a potential cannibal. The change in respiratory-related behavior is thus the result of a change in the overall behavioral strategy adopted under high concentrations of kairomones and kairomones with alarm signal.

In summary, we showed that 1) *I. elegans* larvae recognized conspecific chemical cues as a signal about danger or expected increase in competition; 2) Conspecific alarm signal applied with kairomones increased the intensity of refuge use and immobility in *I. elegans*.; 3) Individuals exposed to conspecific chemical cues balanced food intake and risk avoidance compared to non-exposed individuals, which was observed in behavioral changes.; 4) Oxygen consumption of *I. elegans* did not change in response to conspecific chemical cues. However, *I. elegans* changed their respiratory-related behavior by –increasing the variability of oxygen consumption, which may indicate compensation for the inability to perform respiratory movements other than rectal ones in the face of danger.

## Data Availability

The datasets generated during and/or analyzed during the current study are available from the corresponding author on request (contact: ma.sysiak@student.uw.edu.pl).
